# One-year follow-up of a short specific carbohydrate diet intervention in children with juvenile idiopathic arthritis: A retrospectively controlled study with focus on medical burden

**DOI:** 10.1007/s10067-025-07421-z

**Published:** 2025-04-02

**Authors:** Naima Hagström, Anders Öman, Afsaneh Koochek, Henrik Arnell, Lillemor Berntson

**Affiliations:** 1 Department of Women’s and Children’s Health, Uppsala University, Uppsala, Sweden; 2 Department of Food Studies, Nutrition and Dietetics, Uppsala University, Uppsala, Sweden; 3 Department of Women’s and Children’s Health, Karolinska Institutet, Stockholm, Sweden

**Keywords:** Arthritis, Diet therapy, Juvenile idiopathic

## Abstract

**Objectives:**

Studies on diet as a complementary treatment in children with juvenile idiopathic arthritis (JIA) are limited. We have previously reported initial findings from a study exploring the potential anti-inflammatory effects of a 1-month specific carbohydrate diet (SCD) in children with JIA. This paper presents the full-year follow-up results, primarily focusing on changes in medication needs before and after the intervention.

**Methods:**

Twenty-eight patients with JIA, with low disease activity, were included. The results of disease activity, physical function, pain, morning stiffness, and inflamed joints from the 1-month intervention, as well as long-term effects, were evaluated. The medical burden during the year before and 1 year after the dietary intervention was compared with three times as many retrospective patients with JIA.

**Results:**

Despite adherence challenges, twenty-one children completed at least 1 month on the diet with a significant improvement in clinical variables that appeared to persist for several months. Sixteen children completed a 1-year follow-up, and the medical burden was compared with that of 48 matched retrospective controls. We observed no significant group-level changes in medication use from the dietary intervention. In six participants, the need for medical escalation was eliminated following the dietary intervention, and this effect was maintained for 1 year.

**Conclusion:**

The SCD shows promise in alleviating symptoms in children with JIA, both in the short and long-term. While no significant group-level changes were observed, some participants avoided treatment escalations, suggesting individual benefits. However, larger-scale studies using a less complicated diet are necessary to draw definitive conclusions.

Clinical Trials Identifier NCT04205500, 2019/12/17, retrospectively registered. URL: https://register.clinicaltrials.gov.

**Table:** 

**Key Points** • *The specific carbohydrate diet (SCD) allowed some participants to avoid planned medication escalations, indicating its potential role in managing JIA symptoms.* • *Most participants faced difficulties with long-term adherence to the restrictive SCD, highlighting the need for more sustainable dietary strategies.* • *Further studies are needed to identify specific dietary components that drive benefits and to explore underlying mechanisms for effective dietary recommendations in pediatric rheumatology.*

**Supplementary Information:**

The online version contains supplementary material available at 10.1007/s10067-025-07421-z.

## Introduction

Over the past two decades, major advances have been made in the medical treatment of juvenile idiopathic arthritis (JIA), primarily due to the introduction of biologic antirheumatic drugs (bDMARDs) [1]. While these advances have improved overall outcomes, many patients struggle to achieve remission and continuously experience low-grade disease activity, pain, and fatigue [2–5]. Longitudinal data show that a majority of patients still have an extensive medical burden 18 years after disease onset [3]. Despite advancements in pharmacological treatments, common challenges, such as adverse effects, insufficient efficacy, and injection-related anxiety, highlight the need to explore complementary therapeutic approaches [2, 6, 7].

In recent years, there has been an increasing interest in diet as a potential adjunctive treatment option for JIA among patients and families [8]. This most likely stems from increased knowledge concerning the connection between gut microbiota, metabolites, and the immune system, which can be manipulated through diet [9]. In addition, several identified risk factors for JIA are related to a negative alteration of gut microbiota and intestinal immunity, such as the early administration of antibiotics [10, 11], short duration of breastfeeding or bottle feeding, as well as caesarian section rather than vaginal delivery [11–13]. Indications of negative alterations in the gut microbial community (dysbiosis) have been found in JIA, although the results are not consistent [11, 14–16].

However, despite being ranked as a high research priority by both families and clinicians, dietary intervention studies in children with JIA are limited [8]. In a small pilot study on exclusive enteral nutrition, we have shown that some children with JIA benefit from dietary modification [17]. Building on this, our ongoing research focuses on studying the potential effects of a whole food diet, which we define in this study as a dietary pattern that emphasizes foods in their natural state. For this purpose, we have chosen to examine the specific carbohydrate diet (SCD). While the SCD is mostly known for its demonstrated ability to induce clinical and biochemical remission in inflammatory bowel disease (IBD), it does not necessarily lead to complete healing of the intestinal mucosa [10, 11]. The results in JIA for the first 15 children participating in our study were promising but only reflected the potential effects of the 4-week intervention period [18]. Data on the possible long-term efficacy of the diet are lacking. The SCD can be considered restrictive due to its elimination of many staple foods, including cereal products, rice, and potatoes. This restrictive nature may make long-term adherence challenging [19], raising questions about the sustainability of the SCD as a long-term intervention.

This study aimed to present comprehensive results from the whole cohort of children with JIA who underwent the SCD intervention. We sought to gain a more complete understanding of the potential benefits and sustainability of the SCD in the short and long-term as a potential adjunctive treatment approach for JIA. Our primary objective was to study the need for medical treatment during the year after the intervention, as well as assess the potential changes in medication requirements before and after the intervention compared with matched controls with JIA.

## Methods and materials

### Study design and participants

We conducted an explorative study at the pediatric rheumatology unit of Uppsala University Children’s Hospital in Sweden from September 2017 to January 2021. Children and teenagers with JIA, classified according to the International League of Associations for Rheumatology’s (ILAR) criteria [20], were enrolled after meeting the following inclusion criteria: patients had to be on stable treatment, defined as no changes in medical treatment within the 3 months prior to the study, with the exception of joint injections performed at least 2 months before intervention. Inclusion criteria were mild to moderate disease activity characterized by: (1) no more than two active joints at enrollment, (2) erythrocyte sedimentation rate (*ESR*) ≤ 30 mm/h, and (3) morning stiffness lasting ≥ 15 min, which excludes patients in complete remission [21]. Both the child and the parents had to have the motivation to participate.

### Dietary intervention

The SCD is rich in fruits, vegetables, pulses, fresh meats, poultry, fish, well-fermented dairy products, nuts, and seeds, while excluding grains, starch-rich foods, some additives, and added sugars except for honey. This composition promotes home-cooked meals and minimizes processed food consumption. A more detailed description of the intervention and methods has been published [18].

Participating families received comprehensive SCD information, including a recipe booklet and a list of allowed foods, followed by a dietitian consultation and an inclusion visit at the pediatric rheumatology clinic. The intervention consisted of an optional 2-week preparatory period and a mandatory 4-week strict adherence phase, after which patients could choose to continue or discontinue the diet.

### Control group selection and matching

A population-based cohort of children with JIA (*n* = 292) served as the control group. We took the following steps for comparison between the study group of 16 and the 48 JIA patients in the control group: Stratification of controls based on three key variables: (1) disease category (course type) as per ILAR criteria, (2) gender, and (3) age at onset: 0 to < 5 years, 5 to ≤ 10 years, and > 10 years. Excel was used to randomly select three controls from each stratified subgroup and to match each control with a study participant, ensuring comparability in disease category, gender, and age at onset. To ensure that comparisons were made at equivalent stages of disease progression between cases and controls, an index date was set for the controls to match the disease duration of the corresponding study participant. The index date for the control group was aligned with the first day of the SCD intervention in the study group. Among the 16 participants in the study group, the median index year was 2019 (*IQR* 2018–2020), with a range from 2017 to 2022. For the 48 controls, the median index year was 2020 (*IQR* 2016–2022), with a range from 2012 to 2023.

### Data collection

In the control group, we retrospectively analyzed electronic medical records spanning 2 years—1 year before and 1 year after the index date. We collected monthly data on DMARD and bDMARD treatments for this 2-year period. Due to unreliable data, we excluded NSAID use from our analysis. We also documented all joint injections administered within this 2-year time frame. Data on medication use and number of joint injections were recorded at each visit for the 16 participants in the intervention group and summarized at 1-year follow-up. Corresponding data for the year prior to intervention were retrieved retrospectively from the electronic medical records. This allowed us to compare treatment patterns in the control group with those in the diet intervention group during the year before and after the 1-month dietary intervention. The medication and joint injections during the year prior to the 1-month dietary intervention were also compared with the year post-intervention in the study group (*n* = 16).

### Questionnaires and clinical measures

A comprehensive set of measures was used to evaluate disease activity and physical function at baseline, 2 and 4 weeks of intervention, as well as at 3, 6, and 12 months post-initiation of the intervention. Disease activity was measured using the Juvenile Arthritis Disease Activity Score (JADAS27), which comprises a joint count (0–27 active joints), patient-reported global assessment of well-being on a visual analogue scale (VAS) (0–10 cm) (assessed by a parent if the child is ≤ 9 years old), physician’s global assessment of disease activity on a VAS (0–10 cm), and normalized erythrocyte sedimentation rate ((*ESR* in mm/h—20)/10) on a scale (0–10) with a maximum total score of 57 [22]. Physical function was measured using the child health assessment questionnaire (CHAQ) [23]. In addition, a pain VAS (0–10 cm), the number of minutes of morning stiffness, and the number of active joints were assessed. A physician made a clinical assessment of whether a joint was active or not. At each follow-up visit, patients were asked to rate their level of adherence to the diet.

### Laboratory analyses

Blood samples were collected at each visit. Fecal calprotectin was analyzed at inclusion using a chemiluminescent immunoassay (CLIA; Diasorin Liaison Calprotectin). For patients older than 4 years, a level of < 50 µg/g is considered normal. Vitamin D (S-25-OH) was analyzed using an immunochemistry assay. Reference values for vitamin D status were defined as: < 30 nmol/L, indicating risk of deficiency and 30–50 nmol/L suggesting risk of inadequate status.

### Statistical analysis

Non-parametric tests were employed due to the small sample size and potential non-normal distribution of data. For variables that appeared to be approximately normally distributed, mean, and standard deviation were also calculated. The related-samples Wilcoxon signed-rank test assessed differences in laboratory and clinical variables, with the Hodges-Lehmann related sample analysis estimating confidence intervals for median values. Since age was matched by groups, we used the independent samples median test for comparing age of onset between the intervention group (*n* = 16) and JIA controls (*n* = 48). The independent-samples Mann–Whitney *U* test was used to analyze differences in delta values of joint injections over 2 years between cases and controls, and in disease activity between drop-outs and the 1-year follow-up group. We applied linear regression models with robust standard errors to estimate differences in use of DMARDs and bDMARDs during the year post-intervention between groups, adjusting for pre-intervention use. Statistical significance was set at *p* < 0.05. Analyses were performed using IBM SPSS Statistics for Windows, version 28 (IBM Corp., Armonk, NY, USA). Microsoft® Excel® 2019 MSO (10,396.20023) facilitated randomization of JIA controls, while SAS software (version 9.4, SAS Institute Inc., Cary, NC, USA) was used for the Swimmers plot.

## Results

**Fig. 1:**
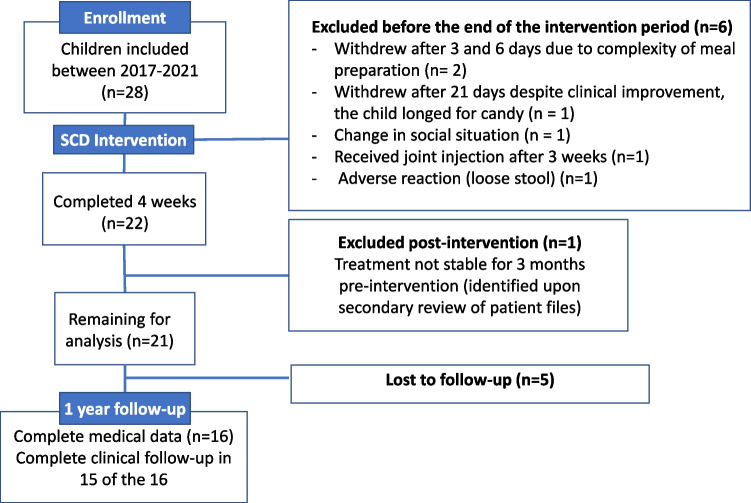
Flow chart showing inclusion, exclusion, and drop-out of participants in an intervention study of the specific carbohydrate diet in children with juvenile idiopathic arthritis

Of the 28 children initially enrolled in the intervention study, seven are excluded for reasons detailed in Fig. 1, one due to a flare. Demographic data on the remaining 21 participants are presented in Table 1. At 1-year follow-up, five participants were lost: two due to COVID-19 restrictions, one had relocated abroad, one cited distance as a barrier, and one withdrew without explanation. The change in JADAS27 scores from the 1-month intervention did not differ between the 16 completers and 5 non-completers (*p* = 0.9). One participant provided incomplete questionnaires at 1 year, resulting in 15 patients for some analyses.

**Table 1:** Demographic data of 21 children with juvenile idiopathic arthritis included in a dietary intervention study with specific carbohydrate diet (*SCD*) for 4 weeks

	Children completing 1 month on SCD *N* = 21	Children completing 1 month on SCD with 1-year follow-up *N* = 16
Gender, girls/boys, *n* (%)	16/5 (76.2/23.8)	12/4 (75/25)
Age at inclusion, Md (*IQR*)	11.7 (9.9–14.5)	11.1 (9.1–13.9)
Age at onset, years Md (*IQR*)	6.1 (3.2–10.1)	6.0 (3.3–9.4)
Disease duration, years Md (*IQR*)	3.1 (2.1–8.0)	3.1 (1.7–6.7)
ILAR category, *n* (%)		
Oligoarticular persistent	11 (52.4)	9 (56.3)
Oligoarticular extended	2 (9.5)	2 (12,5)
Polyarticular RF −	4 (19.0)	4 (25.0)
Enthesitis-related arthritis	2 (9.5)	1 (6.3)
Polyarticular RF +	1 (4.8)	
Juvenile psoriatic arthritis	1 (4.8)	
Medical treatment, *n* (%)		
No medication	7 (33.3)	4 (25.0)
Methotrexate	6 (28.6)	5 (31.2)
TNF-inhibitor + methotrexate	5 (23.8)	4 (25.0)
Tocilizumab.	1 (4.8)	1 (6.2)
Abatacept + methotrexate	1 (4.8)	1 (6.2)
Abatacept	1 (4.8)	1 (6.2)
Laboratory and clinical variables		
Fecal calprotectin at inclusion µg/g Md (min–max)	8.5 (0–70)	
Plasma 25-hydroxyvitamin D at inclusion (nmol/L) Md (min-max) *n* = 18	55.2 (36.4–87.4)	
Plasma 25-hydroxyvitamin D at inclusion compared to at 4 weeks of SCD (nmol/L) *n* = 15	-1.0 (-9.8 - 6.5) *P* = 0.78*	
Weight loss (%) Md (min-max) *n* = 18	2.3 (0.1-7.9)	
Weight gain (%) *n* = 3	0.2, 1.5, 5.8	

*Md* median, *IQR* interquartile range, *ILAR* International League of Associations for Rheumatology, *RF* − rheumatoid factor negative, *RF* + rheumatoid factor positive, *µg/g* microgram/gram, *nmol/L* nanomole/liter

^*^Related-samples Wilcoxon signed-rank test, and related-samples Hodges-Lehman median differences

### Clinical and laboratory findings

Fecal calprotectin did not indicate intestinal inflammation in any patient. Levels of plasma 25-hydroxyvitamin D at inclusion were just below 50 nmol/L in four patients and 34.6 nmol/L in one. One patient received vitamin D supplementation post-intervention. Some of the blood samples collected just before the summer showed low vitamin D status, which normalized in subsequent samples. The 1-month intervention did not significantly affect vitamin D status at group level (Table 1).

### Intervention outcome

The JADAS27, CHAQ, pain VAS, and minutes of morning stiffness improved significantly following the 1-month intervention (Supplementary Fig. 1) (the results for fifteen of these patients have been presented in an earlier publication) [18]. These improvements were sustained for at least 6 months in 15 of the 16 patients at the 1-year follow-up (Table 2) . Of the six patients with one active joint at inclusion, five showed clinical resolution according to clinical judgment within 2–2.5 weeks. One patient developed arthritis in an additional joint after 2.5 weeks.

**Table 2:** Clinical variables in 21 children with juvenile idiopathic arthritis at different time intervals during 1 year after a 4-week dietary intervention

	Four weeks of intervention with SCD			Comparison between inclusion and after 4 weeks of intervention			Time after onset of intervention, months			
	At inclusion	After 2 weeks	After 4 weeks	Median difference	Confidence interval*	*p*-value**	Three	Six	Twelve	*p*-value***
Arthritis, *n* (% of total group)	6 (28.6) *n* = 21	3^a^ (12.5) *n* = 16	2^a^ (4.8) *n* = 21				0 *n* = 15	0 *n* = 15	0 *n* = 15	
JADAS27 (0–57) Md (*IQR*)^b^	4.1 (2.5–8.8) *n* = 21	1.6 (0.8–8.1) *n* = 13	1.9 (0.2–4.2) *n* = 21	− 2.3	(− 3.6)–(− 1.1)	0.003	0.9 (0.5–2.8) *n* = 13	0.7 (0.3–2.4) *n* = 12	1.1 (0.2–3.3) *n* = 15	0.012
CHAQ (0–3) Md (*IQR*)^b^	0.38 (0–0.50) *n* = 21	0.18 (0–0.72) *n* = 16	0.12 (0–0.25) *n* = 21	− 0.19	(− 0.31)–(− 0.002)	0.018	0 (0–0) *n* = 13	0 (0–0.25) *n* = 13	0 (0–0.25) *n* = 15	0.011
Pain VAS (0–100 mm) Md (*IQR*)^b^	23.0 (6–37.0) *n* = 21	4.5 (0.2–41.0) *n* = 16	4.0 (2.0–25.5) *n* = 21	− 11.5	(− 18)–(− 5.5)	0.005	2.0 (0–12.5) *n* = 13	4.0 (2–0–11.0) *n* = 13	4.0 (0–17.0) *n* = 15	0.18
Morning stiffness minutes Md (*IQR*)^b^ (min–max)	15 (0–60) 0–90 *n* = 21	0 (0–11) 0–60 *n* = 16	0 (0–0) 0–40 *n* = 21	− 21	(− 30)–(−10)	< 0.001				

*SCD* specific carbohydrate diet, *JADAS* juvenile arthritis disease activity score, *CHAQ* Child Health Assessment Questionnaire, and *VAS* visual analogue scale

^*^Hodges-Lehman related samples analysis, **Wilcoxon matched-pair signed-rank analysis, *** Wilcoxon matched-pair signed-rank analysis comparing values at baseline with those at 1-year follow-up

^a^Two active joints in one child

^b^Md (*IQR*)

### Medical treatment outcomes

**Fig. 2:**
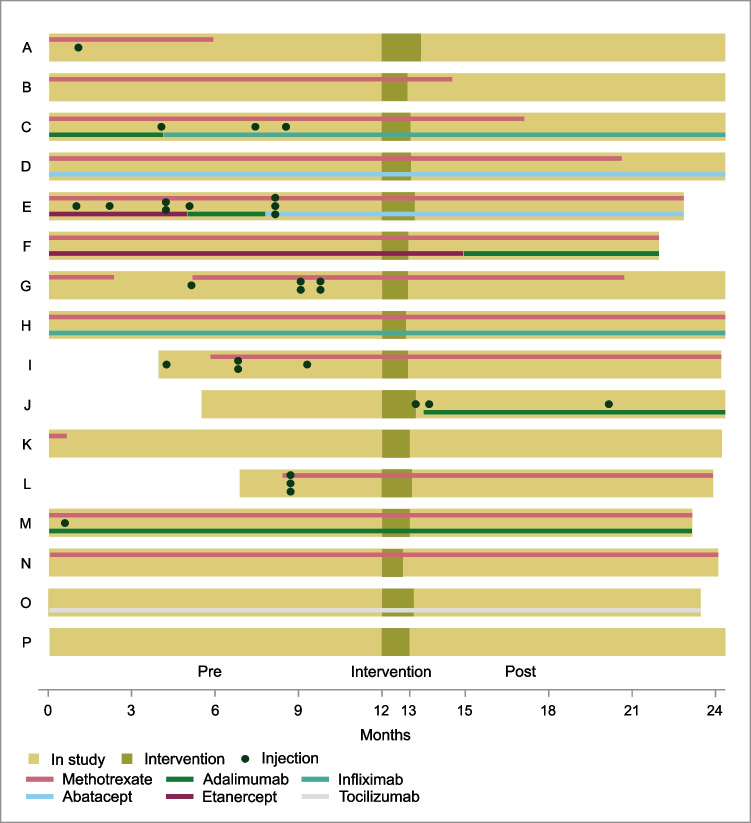
Swimmers plot presenting the individual medical treatment during the months before, during, and after the 1-month intervention with the specific carbohydrate diet

Figure 2 illustrates medical treatments and joint injections in the 16 children during the year before and the year after the intervention. Four patients who were initially on methotrexate, and who required bDMARD addition, opted to try SCD instead and did not require bDMARD in the following year (patients G, I, L, and N). Two patients without initial medical treatment, who required methotrexate, chose SCD and avoided methotrexate use in the subsequent year (patients A, K).

### Retrospective comparison of intervention group and control patients

The 48 control patients with JIA were matched to the intervention group (*n* = 16) based on category of disease, gender, and disease duration, as described. No statistical difference in age of onset was found between the groups (*p* = 0.39) (Table 3) .

**Table 3:** Data on months of medical treatment in 16 patients with JIA during 1 year before and 1 year after a 1-month period of a dietary intervention and in comparison, with 2 years of disease course in 48 children with juvenile idiopathic arthritis (JIA) from our regional study cohort of children with JIA

	Children with JIA treated with SCD for 1 month, *n* = 16			Controls *n* = 48								
	Proportion of treatment		Change	Proportion of treatment		Change	Estimated mean difference in proportion of months with treatment (%) between children treated with SCD and controls					
	The year before	The year after		The year before	The year after				Estimate	95% *CI* lower limit	95% *CI* upper limit	*p*-value*
Treatment with methotrexate												
*Mean*	67.33	54.77	− 12.56	26.06	31.72	5.65	*Mean*	Non-adjusted**	− 18.2	− 37.6	1.1	0.06
*SD*	42.02	45.91	31.24	36.77	43.23	40.59	*Mean*	Adjusted***	− 3.9	− 24.9	17.2	0.72
Min	0	0	− 86.35	0	0	− 91.70						
Max	100	100	30.13	100	100	100						
Treatment with biological agents												
*Mean*	43.70	49.82	6.12	23.23	35.71	12.49	*Mean*	Non-adjusted**	− 6.4	− 20.9	8.1	0.38
*SD*	51.17	51.45	24.35	40.56	46.59	28.21	*Mean*	Adjusted***	− 4.5	− 19.5	10.5	0.55
Min	0	0	− 0.36	0	0	0						
Max	100	100	97.44	100	100	100						

*SCD* specific carbohydrate diet, *SD* standard deviation, *CI* confidence interval

^*^Linear regression model, **non-adjusted for the pre-value, ***adjusted for the pre-value

### Medication use prior to intervention

The intervention group exhibited a higher medical burden in the year before inclusion compared with the control group. Specifically, methotrexate use was significantly higher in the intervention group, with a median of 100 (*IQR* 37.5–100) months compared with a median of 0 (*IQR* 0–50) months in the controls (*p* < 0.001). For use of biological agents, the intervention group had a median of 50 (*IQR* 0–100) months, while the control group had a median of 0 (*IQR* 0–29) months, with a *p*-value of 0.054.

When comparing medication use from the year before with the year after the intervention, no statistical difference was observed between the intervention group and the JIA controls, but there was a non-significant tendency for methotrexate use (Table 3). The mean number of joint injections in the intervention group was 1.5 during the year before the intervention and decreased to 0.19 the year after. In contrast, the control group had means of 0.88 and 0.35 for the respective years. The change in the number of joint injections administered during the 2 years did not show a statistically significant difference when comparing the two groups (*p* = 0.20).

### Sustainability of the SCD-treatment

Adherence to the SCD varied among the participants. Two patients adhered strictly to the SCD throughout the entire year of observation. Among the 16 patients who could be followed for 1 year, families reported approximately 81% adherence at 2 months post-intervention, which decreased to 50% at 3 months, and further declined to 25% at 6 months. After 1 year, five children continued to use the SCD to some extent, with the majority reporting that they still avoided regular snacks, sweets, and fast food.

## Discussion

After a 1-month dietary intervention, the positive outcomes observed in our initial cohort of 15 children were further supported when the total sample size had increased to 21 participants. Specifically, we noted significant reductions in JADAS27 scores, enhanced physical function, decreased pain, and shortened duration of morning stiffness. Importantly, these preliminary results indicate that clinical benefits were sustained for several months post-intervention in the follow-up group. Comparison with a matched control group of the medical burden before and after the intervention revealed no significant differences in long-term medication requirements; several patients who were candidates for treatment escalation did not require planned changes in their regimens’ post-intervention, suggesting clinically meaningful effects for some individuals.

Interestingly, clinical improvements seemed to be sustained over time, despite the fact that most participants fully adhered to the SCD for only a few months. This observation challenges our current understanding, since previous research indicates rapid changes in fecal microbiota following dietary modifications [24]. Several hypotheses may explain this result: it is possible that dietary changes could have more profound functional effects than what can be observed in fecal microbiota alone, or the minor dietary modifications maintained by participants long-term might have been sufficient to sustain the positive effects.

The primary outcome of medication usage showed mixed results. While there was no significant change at group level, six participants who previously needed the addition of DMARD or bDMARD to their treatment plan, no longer required this escalation. This discrepancy may be attributed to the small sample size, limiting the power to detect significant group-level changes. Nevertheless, these individual cases suggest that dietary modifications may affect disease control, potentially reducing the need for medication escalation, at least in some patients.

The short full adherence period likely relates to the challenges associated with the restrictive nature of the diet, as previously reported [19]. Weight loss, a known risk with restrictive diets, was observed in the majority (*n* = 18) following the 1-month intervention, while three gained weight. The more pronounced weight loss in some children may have contributed to the observed anti-inflammatory effect [25]. Adherence difficulties, along with the sustained clinical improvements despite limited long-term compliance, raise important questions about the optimal strictness of the diet and which specific foods drive the results.

While the specific components of SCD driving improvement remain unknown, previous research suggest that the richness of vitamins, minerals, dietary fiber, and other bioactive components from fruits and vegetables likely had a positive impact [26, 27]. Dietary fiber is especially important for colon bacteria in relation to the production of short-chain fatty acids (SCFAs), which have profound immunological effects, for example, stimulating the production of T-regulatory cells in the colon [28–30]. However, unpublished data reveal that participants in our study cohort consumed more fruit and vegetables compared to the national average, as reported in a Swedish national dietary survey [31].

The SCD’s exclusion of saccharose may also have contributed to possible effects. Studies in adults with RA have shown that the consumption of sweets and sugar-sweetened beverages may increase inflammation [32, 33]. Additionally, processed foods, soft drinks and sodas often contain high amounts of exogenous advanced glycation end products (AGEs), shown to cause oxidative stress and inflammatory reactions, which is negative for overall health, particularly for individuals with autoimmune diseases [34].

Some components of the SCD for treating children with JIA need further investigation. For instance, the exclusion of high-starch foods contradicts evidence suggesting that various polysaccharides, including starch, can positively affect intestinal health [35]. Similarly, while gluten is strictly excluded in the SCD, there is no study on whether following a solely gluten-free diet improves inflammatory activity in JIA; the results in adults with RA are not conclusive [36, 37]. In summary, there is insufficient evidence to recommend a gluten-free diet in RA [38] and recommendations for JIA are lacking.

This study had several limitations, including a high drop-out rate, resulting in a small study cohort, far too small for analysis of differences between disease categories. However, the majority of participants (81%) belonged to the oligoarticular persistent, oligoarticular extended and poly RF^−^ categories, sharing clinical similarities and HLA associations [39]. The strict inclusion criteria limited the number of eligible patients for possible inclusion, since a child could not be too inflammatorily active for 3 months before inclusion. Any change in medication during the 3 months prior to or during the intervention excluded the child so as not to impact the results. Another limitation was the use of self-reported data, which may have introduced bias.

Our initial study design was enhanced by incorporating data from a regional cohort as a control group, allowing for more robust conclusions regarding pharmacological treatment outcomes. While this addition strengthened our analysis, it is important to note that the observation period for a small subset of controls extended up to 5 years earlier than the study group. However, the majority of patients had comparable observation periods. Despite these improvements, we acknowledge the potential impact of this temporal variation on our results. The control group was randomly selected and carefully matched for factors that might affect both disease course and the microbiota composition, including gender, age of onset, and disease category [40–42]. Since disease activity decreases during the first few years in most JIA trajectories, this supported our decision to use an index date in the control group corresponding to the intervention date, enabling meaningful comparisons between groups [43]. The patients in the intervention group had a higher burden of medical treatment prior to the intervention compared with the controls, likely influencing the comparisons. The high medical burden in the intervention group may also have contributed to inclusion bias due to a higher motivation to participate in a dietary intervention.

Although children with JIA may not have identified major problems in their intestinal mucosa/immune system, there are indications for a disrupted mucosal integrity [44]. We speculate that a diet such as the SCD may improve this disruption by influencing the metabolic function of the microbiota, potentially playing a positive immunological role in JIA. However, the support for some of the components in SCD is weak or lacking, and identifying specific beneficial dietary elements should be a focus in further research.

## Conclusion

This study, which explored the impact of the SCD on medication requirements in children with JIA, yielded mixed results. While no significant group-level changes in medication use were observed, some participants avoided planned escalations, indicating potential individual benefits. Clinical improvements appeared to persist despite adherence challenges, suggesting a potential benefit from partial dietary modifications. However, while our findings are promising, they should be interpreted cautiously due to the study’s small sample size. Larger, more robust studies are needed to clarify the role of dietary interventions for children with JIA. Nevertheless, this research opens new avenues for exploring dietary interventions as an adjunctive strategy in JIA treatment. Future research should focus on identifying the key dietary components driving the beneficial effects so more sustainable and targeted nutritional approaches for JIA management can be developed. As our understanding of the diet-inflammation-autoimmune relationship grows, integrating nutrition into comprehensive care plans for children with JIA may become an increasingly valuable strategy for improving long-term outcomes and quality of life.

## Supplementary Information

Below is the link to the electronic supplementary material.

Supplementary file1 (PDF 256 KB)

## Data Availability

The datasets generated and/or analyzed during the current study are not publicly available for ethical and privacy reasons but are available from the corresponding author on reasonable request.

## Abbreviations

JIA: Juvenile idiopathic arthritis
RA: Rheumatoid arthritis
SCFAs: Short-chain fatty acids
SCD: Specific carbohydrate diet
CD: Crohn’s disease
DMARD: Disease-modifying anti-rheumatic drug
bDMARD: Biological DMARD
ESR: Erythrocyte sedimentation rate
CHAQ: Child health assessment questionnaire
JADAS: Juvenile arthritis disease activity score
VAS: Visual analogue scale
NSAIDs: Non-steroidal anti-inflammatory drugs
