# Supplementary figures and images for: One-year follow-up of a short specific carbohydrate diet intervention in children with juvenile idiopathic arthritis: A retrospectively controlled study with focus on medical burden
Source: Clin Rheumatol. 2025 Apr 2;44(5):2031–41. doi: 10.1007/s10067-025-07421-z (PMC12078398; doi:10.1007/s10067-025-07421-z)

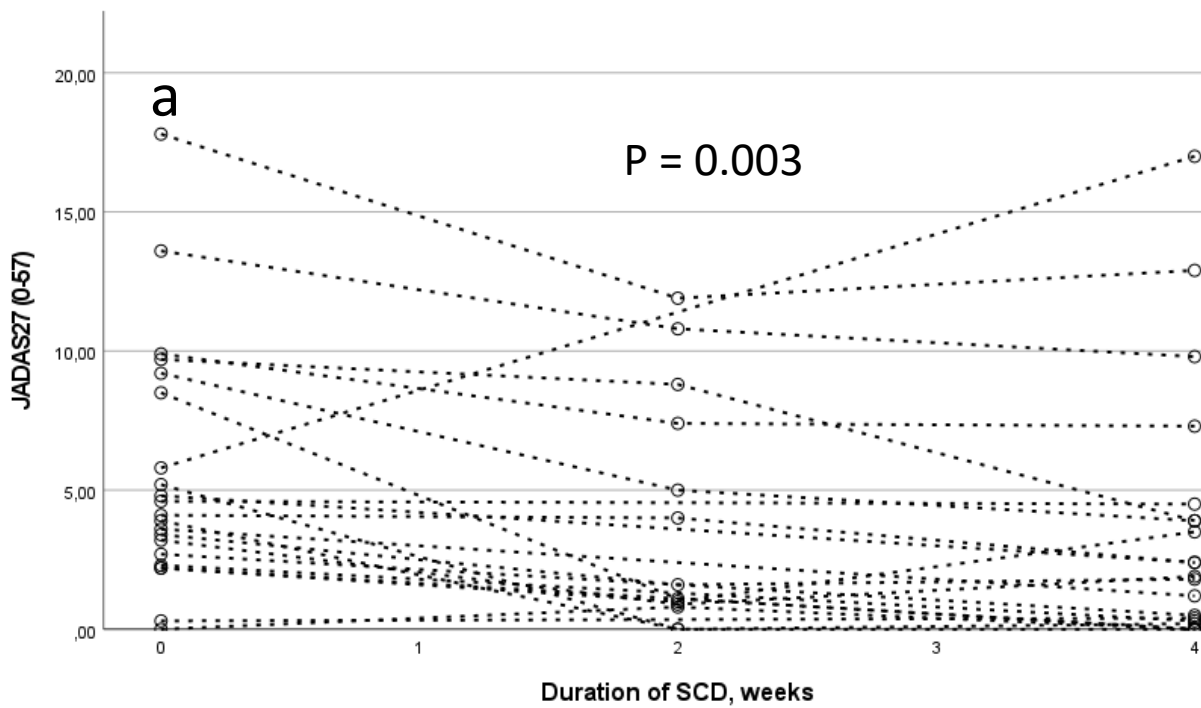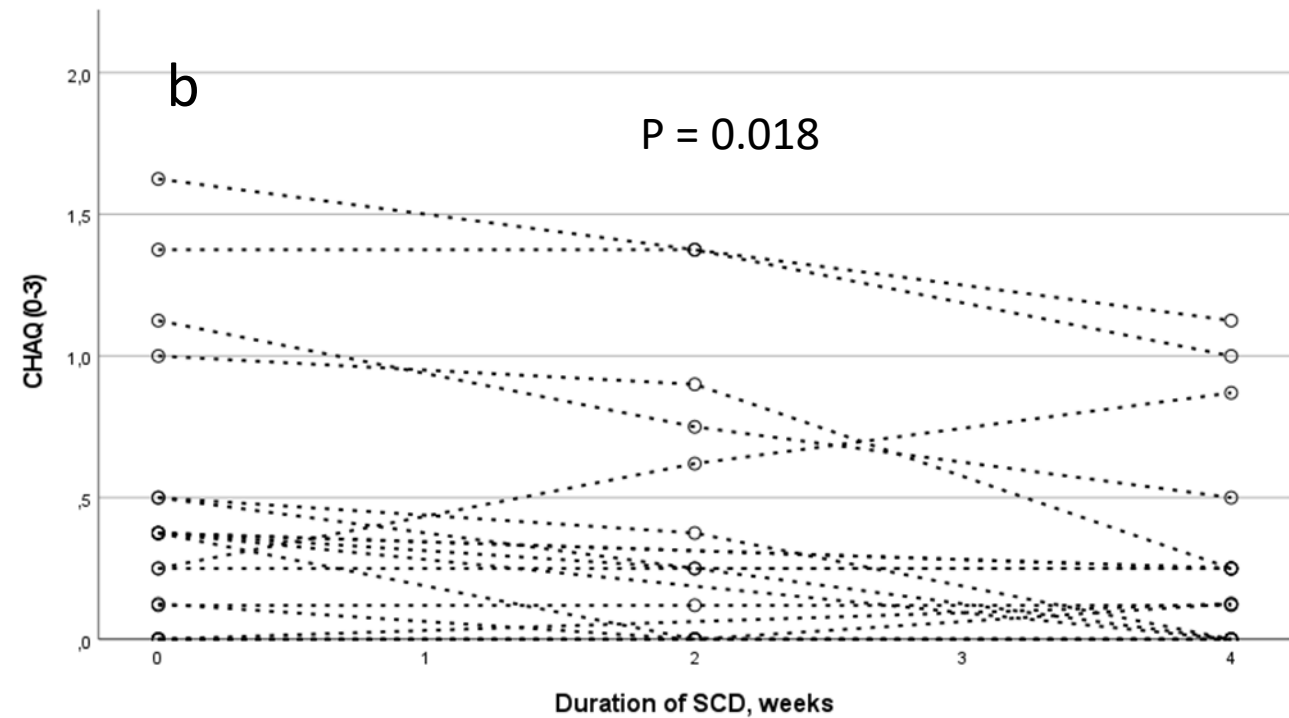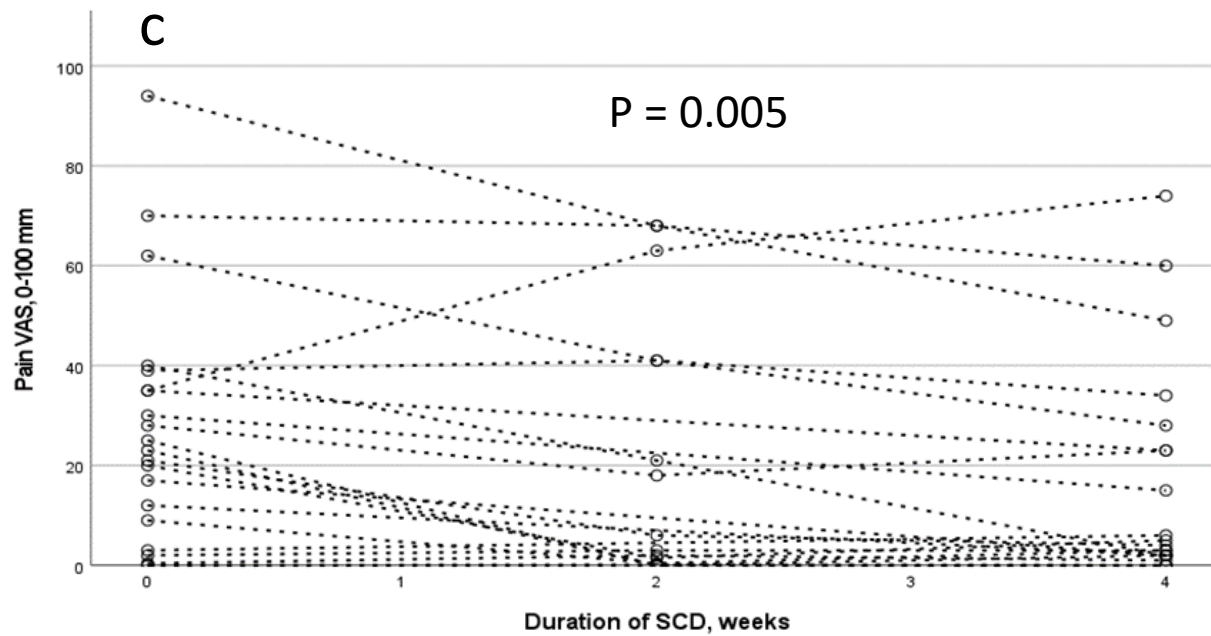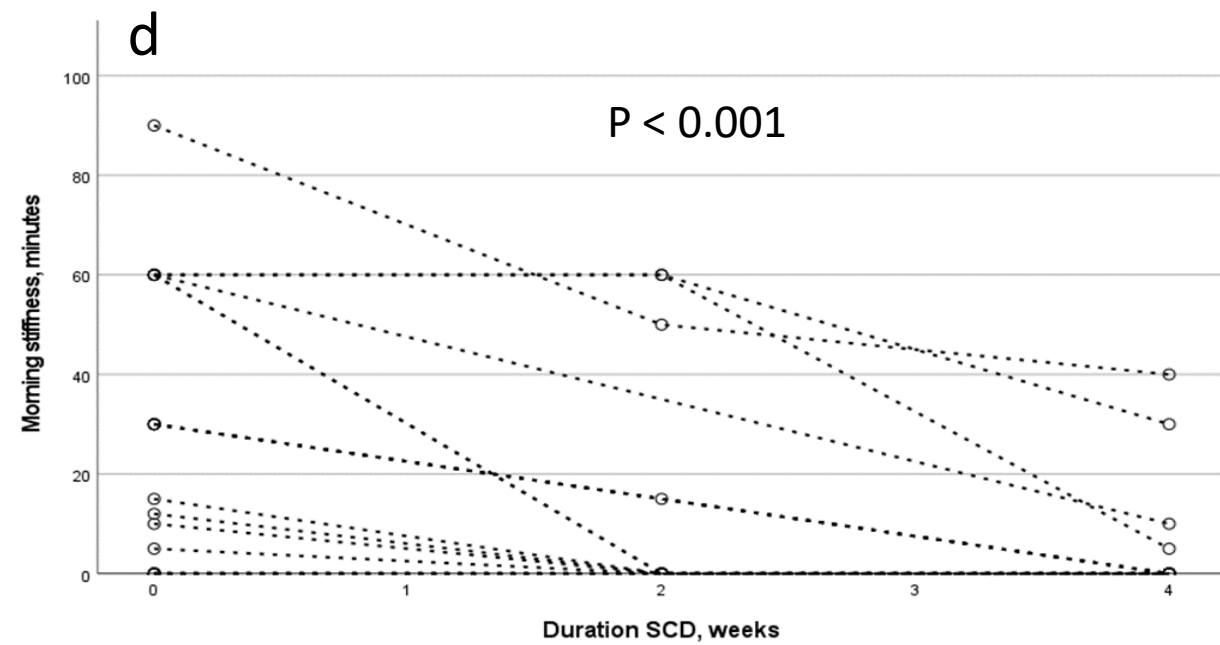

Supplement: Supplementary file 1 — Supplementary file1 (PDF 256 KB) [file 10067_2025_7421_MOESM1_ESM.pdf]
